# Agaricales Mushroom Lignin Peroxidase: From Structure–Function to Degradative Capabilities

**DOI:** 10.3390/antiox10091446

**Published:** 2021-09-12

**Authors:** María Isabel Sánchez-Ruiz, Iván Ayuso-Fernández, Jorge Rencoret, Andrés Manuel González-Ramírez, Dolores Linde, Irene Davó-Siguero, Antonio Romero, Ana Gutiérrez, Angel T. Martínez, Francisco Javier Ruiz-Dueñas

**Affiliations:** 1 Centro de Investigaciones Biológicas “Margarita Salas” (CIB), Consejo Superior de Investigaciones Científicas (CSIC), 28040 Madrid, Spain; marisasr@cib.csic.es (M.I.S.-R.); ivan.ayuso-fernandez@nmbu.no (I.A.-F.); amgonram@gmail.com (A.M.G.-R.); lolalinde@cib.csic.es (D.L.); ireneids3@gmail.com (I.D.-S.); romero@cib.csic.es (A.R.); 2 Instituto de Recursos Naturales y Agrobiología de Sevilla (IRNAS), Consejo Superior de Investigaciones Científicas (CSIC), 41012 Seville, Spain; jrencoret@irnase.csic.es (J.R.); anagu@irnase.csic.es (A.G.)

**Keywords:** Agaricales, lignin peroxidase, catalytic tryptophan, crystal structure, transient-state kinetics, reduction potential, model dimers, non-phenolic lignin, lignosulfonate degradation, NMR spectroscopy

## Abstract

Lignin biodegradation has been extensively studied in white-rot fungi, which largely belong to order Polyporales. Among the enzymes that wood-rotting polypores secrete, lignin peroxidases (LiPs) have been labeled as the most efficient. Here, we characterize a similar enzyme (ApeLiP) from a fungus of the order Agaricales (with ~13,000 described species), the soil-inhabiting mushroom *Agrocybe pediades*. X-ray crystallography revealed that ApeLiP is structurally related to Polyporales LiPs, with a conserved heme-pocket and a solvent-exposed tryptophan. Its biochemical characterization shows that ApeLiP can oxidize both phenolic and non-phenolic lignin model-compounds, as well as different dyes. Moreover, using stopped-flow rapid spectrophotometry and 2D-NMR, we demonstrate that ApeLiP can also act on real lignin. Characterization of a variant lacking the above tryptophan residue shows that this is the oxidation site for lignin and other high redox-potential substrates, and also plays a role in phenolic substrate oxidation. The reduction potentials of the catalytic-cycle intermediates were estimated by stopped-flow in equilibrium reactions, showing similar activation by H_2_O_2_, but a lower potential for the rate-limiting step (compound-II reduction) compared to other LiPs. Unexpectedly, ApeLiP was stable from acidic to basic pH, a relevant feature for application considering its different optima for oxidation of phenolic and nonphenolic compounds.

## 1. Introduction

Plant biomass is an abundant renewable resource that has attracted an increasing interest during the last decades in the context of lignocellulose biorefinery [1,2]. It is largely made up of the polysaccharides cellulose and hemicellulose, and the aromatic polymer lignin [3]. While cellulose is used in the pulp and paper industry and in the production of added value compounds and biofuels, lignin has been underused, mainly due to its recalcitrance [4,5]. For this reason, organisms able to modify lignin in nature have been widely studied in the last decades, either for a greener degradation of the polymer or in the production of added-value aromatic compounds [6,7,8,9,10].

Although some bacteria [11,12] and soft rot fungi [13] are also involved in lignin degradation acting on the phenolic moiety and degradation products, white-rot fungi are the only organisms able to extensively mineralize native lignin [9,14,15]. They use an array of oxidative enzymatic tools for its extracellular degradation, among which the ligninolytic peroxidases play a major role [9,16]. These enzymes are part of class-II of the peroxidase–catalase superfamily [17] and, according to the oxidation sites in their molecular architecture, are classified into three families [16]: (i) manganese peroxidases (MnPs) harboring a Mn-binding site where Mn^2+^ is oxidized to Mn^3+^, which acts as a diffusible mediator both oxidizing the minor phenolic moiety of lignin and generating strong oxidizers by initiating lipid peroxidation [18] (members of the short MnP subfamily are also able to oxidize phenolic compounds in direct contact with the heme cofactor [19]); (ii) lignin peroxidases (LiPs) containing a solvent-exposed catalytic tryptophan where they directly oxidize high redox-potential non-phenolic aromatic substrates [20,21]; and (iii) versatile peroxidases (VPs) accommodating in a single enzyme the catalytic sites previously described for MnPs and LiPs [22]. In the evolution of Polyporales, where most white-rot fungi are included, MnPs are ancient enzymes from which the other two families arose, first by incorporating a solvent-exposed tryptophan generating VPs, and later by losing the Mn-binding site generating the LiPs that we can find today together with more ancient families [19,23].

LiPs are the most efficient enzymes oxidizing lignin, being isoenzyme H8 from the white-rot fungus *Phanerochaete chrysosporium* [16] corresponding to LiPA from its sequenced genome [24], the best known ligninolytic peroxidase. This enzyme has been extensively studied from different viewpoints, demonstrating its capacity to oxidize non-phenolic lignin directly [25,26,27]. Since its discovery [28], different LiP and LiP-like enzymes have been characterized (see family AA2 in CAZY database, http://www.cazy.org/AA2.html; accessed on 16 August 2021), but only in white-rot Polyporales. However, the recent investigation of dozens of genomes in species of Agaricales have revealed that LiP genes also appeared in this fungal order, through an evolutionary pathway that is different from the one that gave rise to the Polyporales LiPs [29], indicating that they may also play an important role in other lignocellulose degrading fungi. Here, we characterize the first of these peroxidases described in Agaricales, the LiP of the grass-litter mushroom *Agrocybe pediades* (ApeLiP herein after), whose genome has been recently sequenced. With this purpose, we cloned the ApeLiP gene in *Escherichia coli*, overexpressed the protein in the form of inclusion bodies, and after enzyme activation and purification, we investigated the structural and functional properties of the new peroxidase, including its degradative capabilities.

## 2. Materials and Methods

### 2.1. Gene Synthesis and Site-Directed Mutagenesis

The coding DNA sequence of the ApeLiP gene (https://mycocosm.jgi.doe.gov/Agrped1/Agrped1.home.html, accessed on 16 August 2021; JGI #705809) was synthesized by ATG biosynthetics (Merzhausen, Germany) after codon optimization for *E. coli* expression using OPTIMIZER [30]. The synthesized DNA sequence was cloned into the *Nde*I and *BamH*I restriction sites of the expression vector pET23b(+) (Novagen, Merck, Darmstadt, Germany). *E. coli* DH5α was selected for plasmid propagation.

The W166A mutation was introduced by PCR using the whole pET23b-ApeLiP plasmid as a template, and forward (5′-CGTTACCGCCGAAGTGATTGCTCTGTTGGCA TCGCACTCC-3′) and reverse (5′-GGAGTGCGATGCCAACAGAGCAATCACTTCGGC GGTAACG-3′) primers (the mutated codon is shown underlined) which were designed complementary to opposite strands of the same DNA region. PCR reactions were carried out in an Eppendorf Mastercycler Pro S using 10 ng of template DNA, 250 μM each dNTP, 125 ng of direct and reverse primers, 2.5 units of *Pfu* Turbo DNA polymerase AD (Stratagene, San Diego, CA, USA) and the manufacture’s reaction buffer. Reaction conditions were as follows: (i) a “hot start” of 95 °C for 1 min; (ii) 18 cycles at 95 °C for 50 s, 55 °C for 50 s, and 68 °C for 10 min; and (iii) a final cycle at 68 °C for 10 min. Template DNA was cleaved with *Dpn*I (Roche, Basel, Switzerland) and remaining salts from PCR were eliminated using a PCR purification kit (Qiagen). *E. coli* DH5α cells were transformed with the resulting plasmid and grown in LB-ampicillin (100 µg/mL) agar plates. Plasmid purification was carried out using the High Pure Plasmid Isolation kit (Roche, Basel, Switzerland) and introduction of the mutation was confirmed by sequencing.

### 2.2. Heterologous Expression and In Vitro Activation

For recombinant protein production, plasmids containing the coding DNA sequences for ApeLiP and its W166A variant were transformed into *E. coli* BL21 (DE3)pLysS. Overnight cultured cells in LB-ampicillin (100 µg/mL) medium, with 34 µg/mL chloramphenicol, were diluted (1:100) in 1 L of Terrific Broth (TB) [31] containing both antibiotics, and grown at 37 °C and 200 rpm until OD_600nm_~0.6. Cells were induced with 1 mM isopropyl β-D-1-thiogalactopyranoside for 4 h, and harvested by centrifugation at 7000 rpm and 4 °C for 5 min. Bacterial pellets were resuspended in lysis buffer (50 mM Tris-HCl, pH 8.0, supplemented with 10 mM EDTA, 5 mM dithiothreitol [DTT] and 2 mg/mL lysozyme) and treated with 0.1 mg/mL of DNaseI (Roche, Basel, Switzerland) at 4 °C for 30 min. After cell disruption by sonication, and centrifugation at 15,000 rpm and 4 °C for 30 min, the supernatant was discarded and the insoluble fraction, where protein inclusion bodies accumulated, was preserved. Protein aggregates were washed and solubilized in 8 M urea as described for other fungal oxidoreductases [32,33].

Once solubilized, a factorial screening of conditions for enzyme in vitro folding was performed. Small-scale assays were carried out using 200 μL folding-mixture volumes in 96-well microplates, where the concentration of the components and folding conditions were modified. These included urea (0.16–2.8 M), oxidized glutathione (GSSG) (0–1.6 mM), hemin (5 or 15 μM), pH (7–9.5), temperature (4 or 25 °C), time (24–72 h) and, in the case of the W166A variant, glycerol (0 or 20%). All folding mixtures were prepared using constant protein (0.1 mg/mL), DTT (0.1 mM), EDTA (0.02 mM), buffer (50 mM Tris-HCl) and CaCl_2_ (5 mM) concentrations. Refolding efficiency was checked by measuring activity towards 5 mM 2,2′-azinobis(3-ethylbenzothiazoline-6-sulfonate) (ABTS), in 100 mM sodium tartrate, pH 3.0, containing 0.1 mM H_2_O_2_. Optimal conditions for recombinant ApeLiP folding were 0.16 M urea, 0.8 mM GSSG and 15 μM hemin in 50 mM Tris-HCl, pH 9, and 24 h incubation at 4 °C. The recombinant W166A variant was folded under the same conditions but using 5 μM hemin and 20% glycerol, in 50 mM Tris-HCl, pH 7.5.

### 2.3. Protein Purification and Quantification

For protein purification, refolding mixtures were 40-fold concentrated (Pellicon and Amicon systems, with 10-kDa cut-off membranes, from Merck, Darmstadt, Germany; and Cole-Parmer, Vernon Hills, IL, USA, respectively) and ultracentrifuged (35,000 rpm, 4 °C, 1 h) for glycerol elimination, when present. Soluble fractions were dialyzed against 20 mM sodium acetate, pH 4.0, supplemented with 1 mM CaCl_2_, at 4 °C for 3 h to induce misfolded protein precipitation, centrifuged at 8000 rpm and 4 °C for 15 min, and re-dialyzed in 20 mM sodium acetate, pH 5.5, containing 1 mM CaCl_2_ at 4 °C. Proteins were finally loaded into a 6-mL Resource-Q column (GE-Healthcare, Chicago, IL, USA) and eluted with a 0–500 mM NaCl gradient, at 2 mL/min flow, in 20 mM sodium acetate, pH 5.5, containing 1 mM of CaCl_2_. Protein purification was confirmed by SDS-PAGE in 12% polyacrylamide gels with 1% mercaptoethanol using Precision Plus Protein Dual Color Standards (Bio-Rad, Hercules, CA, USA) and Coomassie R-250 staining. UV-visible spectra of the purified proteins were recorded in a Cary4000 spectrophotometer. Molar extinction coefficients of ApeLiP (169,000 M^−1^·cm^−1^) and its W166A variant (163,500 M^−1^·cm^−1^) were calculated according to Lambert Beer’s law, by measuring enzyme absorbance at 410 nm and referring to protein concentration calculated as the average of the values obtained with NanoDrop (NanoDrop 2000 spectrophotometer; Thermo Fisher Scientific, Waltham, MA, USA), Qubit (Invitrogen Qubit 3.0 Fluorometer; Thermo Fisher Scientific, Waltham, MA, USA), and pyridine hemochrome assay [34].

### 2.4. ApeLiP Crystallization, Data Collection, and Refinement

Crystallization of native ApeLiP was achieved with the sitting-drop vapor diffusion method at 295 K using a Cartesian Honeybee System (Genomic Solutions, Ann Arbor, MI, USA). Initial screening was conducted in 96-well sitting drop plates (Swissci, MRC) with two different crystallization kits (JBScreen Classic from Jena Bioscience, Jena, Germany; and Wizard Classic I-IV from Molecular Dimensions, Sheffield, UK). Each crystallization drop consisted of 0.2 µL of protein solution (10 mg/mL, in 20 mM sodium acetate, pH 5.5, containing 1 mM of CaCl_2_) and 0.2 µL of precipitant, equilibrated with 50 µL of the reservoir solution. Crystals were obtained in 100 mM MES (pH 6.5), 25% PEG 550 MME and 10 mM ZnSO_4_ after three weeks. The crystals in cryoprotectant solution, containing the mother liquor supplemented with 25% (*v*/*v*) PEG 400, were frozen in liquid N_2_ prior to data acquisition.

A complete data set was collected at the BL13 XALOC beamline of the ALBA Synchrotron (Cerdanyola del Vallès, Spain). Data were processed using XDS [35] and merged and scaled with AIMLESS [36], from the CCP4 package. The structure was solved by molecular replacement using the crystal structure of *Pleurotus ostreatus* VP (4BLN) as the search model and the program PHASER implemented in the CCP4 package [37]. The initial model was refined using REFMAC [38] and alternating manual rebuilding with COOT [39]. The final model was analyzed and validated using MolProbity [40].

### 2.5. pH and Temperature Stability

The pH stability was estimated by incubating the purified enzymes (1 µM) in the range of pH 2–10, using 100 mM Britton–Robinson buffer at 25 °C. Residual activities were measured at 0, 4, and 24 h using 10 nM enzyme and 1.25 mM ABTS as substrate in 100 mM sodium tartrate, pH 3.0, with 0.1 mM H_2_O_2_. For each enzyme, the highest activity after 1 min (at any pH) was taken as 100%, and the residual activities at the different time and pH conditions were provided as percentages of this maximal value.

Thermal stability of ApeLiP was studied by measuring both the residual activity and secondary structure loss after incubation at different temperatures. To study the effect on enzyme activity, ApeLiP (1 µM) was incubated in 10 mM sodium acetate, pH 5.5, in the range of 25–85 °C for 10 min. Residual activity was determined at 25°C using 10 nM enzyme and 1.25 mM ABTS as described above. Temperature stability was presented as the 10 min T_50_ value, i.e., the temperature at which 50% of the activity was lost after 10 min incubation. 

The effect of temperature on protein denaturation was studied by circular dichroism (CD) from 25 to 70 °C at 30 °C/h using a J-720 spectropolarimeter (Jasco, Oklahoma City, OK, USA) equipped with a temperature controller and a thermostated cell holder. Samples containing 11 μM enzyme in 20 mM sodium acetate, pH 5.5, and 1 mM CaCl_2_ were measured using a cell with 0.1 mm optical path length. The thermal melting profile was represented, and T_m_ value was calculated as the temperature at the midpoint of the unfolding transition.

### 2.6. Steady-State Kinetic Constants 

Five substrates were selected for the first kinetic characterization of ApeLiP: (i) ABTS is oxidized yielding the cation radical ABTS^·+^ (ε_436_ 29,300 M^−1^·cm^−1^); (ii) 2,6-dimethoxyphenol (DMP) once oxidized dimerizes to form the colored product coerulignone (ε_469_ 55,000 M^−1^·cm^−1^); (iii) veratryl (3,4-dimethoxybenzyl) alcohol (VA) is oxidized to veratraldehyde (ε_310_ 9300 M^−1^·cm^−1^); (iv) Reactive Black 5 (RB5) is decolorized (ε_598_ 30,000 M^−1^·cm^−1^); and (v) Mn^2+^ oxidation forms Mn^3+^–tartrate complex (ε_238_ 6500 M^−1^·cm^−1^). Absorbance changes during substrate oxidation were followed using a Thermo Scientific Biomate5 spectrophotometer, at 25 °C, optimal pH, and saturating H_2_O_2_ concentration. Before kinetic analyses, optimal pH for each substrate was determined in the range of pH 2–10 using 10–100 nM enzyme and 1.25 mM ABTS, 6 mM DMP, 50 µM RB5 and 100 mM VA, in the case of the wild-type enzyme; and 5 mM ABTS and 2 mM DMP in the case of the W166A variant. All reactions were conducted in 100 mM Britton–Robinson buffer with the addition of 0.1 mM H_2_O_2_. To determine the H_2_O_2_ optimal concentrations, kinetic constants were analyzed using ABTS (5 mM) as reducing substrate in 100 mM sodium tartrate, pH 3.0. Kinetic parameters were obtained by fitting the data to: (i) the Michaelis–Menten equation *v* = (*k*_cat_ [S])/(*K*_m_ + [S]); (ii) the equation describing inhibition *v* = (*k*_cat_[S])/(*K*_m_ + [S] + (1 + ([S]/*K*_i_))); or (iii) the Hill equation *v* = (*k*_cat_[S]^n^)/(*K*_m_^n^ + [S]^n^), using SigmaPlot software. Double kinetic curves obtained for ApeLiP oxidation of ABTS enabled calculation of two sets of kinetic constants, corresponding to low and high efficiency oxidation sites [41].

Reactions with lignin model dimers were also carried out. Oxidation of phenolic guaiacylglycerol-β-guaiacyl ether (GGE) and non-phenolic veratrylglycerol-β-guaiacyl ether (VGE) were followed over time by analyzing spectral changes with an Agilent 8453 UV-visible diode-array spectrophotometer in a wavelength range of 200 to 800 nm. With this purpose, 1 mM VGE or 0.25 mM GGE were mixed with 0.8 µM and 0.1 μM enzyme, respectively, at optimal pH 3. Reactions were started by H_2_O_2_ addition (0.4 mM) and followed for 5 and 10 min for GGE and VGE, respectively. Oxidation of GGE was followed by absorbance loss (ε_275_ 4400 M^−1^·cm^−1^) while oxidation of VGE was followed by absorbance increase due to veratraldehyde formation (ε_310_ 9300 M^−1^·cm^−1^).

### 2.7. Native and Acetylated Softwood and Hardwood Lignins

Softwood (*Picea abies*) and hardwood (*Eucalyptus grandis*) lignosulfonates provided by G.E. Fredheim (Borregaard AS, Sapsborg, Norway) were used to study the ligninolytic capabilities of ApeLiP. Lignosulfonates were prepared as previously described [26], by dialyzing them first in 50 mM Tris-HCl, pH 8.0, containing 10-mM EDTA, and then in Milli-Q water twice.

Acetylation of lignosulfonates (50 mg) was performed in a 50-mL flask with 3 mL of pyridine–acetic anhydride (1:1, *v*/*v*) stirred for 24 h at room temperature. Then, 10 mL of aqueous methanol (50%) were added, and the mixture was evaporated to dryness under vacuum. The process was repeated with toluene (3 × 10 mL), and methanol (1 × 10 mL). Finally, the acetylated lignosulfonates (60–65 mg) were dried at 50 °C overnight.

### 2.8. Estimation of Transient-State Kinetic Constants with Lignosulfonates

Formation of Compound I (CI), Compound II (CII), and resting-state (RS) enzyme intermediates (Figure S1) was followed using a stopped-flow rapid spectrophotometry equipment (Bio-Logic, Seyssinet-Pariset, France) synchronized with a TIDAS diode array detector (J&M Analytik, Essingen, Germany), and the BioKine software (Bio-Logic, Seyssinet-Pariset, France). All experiments were made in 100 mM sodium tartrate, pH 3.0, at 25 °C. For CI formation kinetics, ApeLiP (1 µM final concentration) was mixed with different concentrations of H_2_O_2_ (0.5–20 molar equiv) and the reaction was followed at 397 nm as previously described for other ligninolytic peroxidases [42,43]. Pseudo first-order rate constants (*k*_1obs_) were calculated from the single-exponential traces observed. Plot of *k*_1obs_ *vs.* H_2_O_2_ concentration was fitted to a linear equation from which the apparent second-order rate constant (*k*_1app_) for ApeLiP reaction with H_2_O_2_ was calculated.

For analyzing the rate-limiting step in ApeLiP reaction with lignin, CII formation was ensured by mixing a solution of RS enzyme containing 1 equiv of potassium hexacyanoferrate(II) (ferrocyanide) with 2.5 equiv of H_2_O_2_ for 2 s. Then, CII reduction to RS was measured at 410 nm (the maximum of ApeLiP RS) upon mixing with different concentrations of acetylated and non-acetylated softwood and hardwood lignosulfonates. Representations of the pseudo-first-order rate constants calculated from the single-exponential traces at 410 nm (*k*_3obs_) *vs.* lignosulfonate concentration were fitted to either linear or hyperbolic equations. For those fitting to linear models, only an apparent second-order rate constant for CII reduction (*k*_3app_) was calculated. Nonlinear least-squares fitting to the hyperbolic model allowed to obtain mean values and 95% confidence intervals for the CII-lignin complex dissociation constant (*K*_D3_) and the first order rate constant (*k*_3_) of this reaction step. Fitting of these constants to the normalized equation: k_3obs_ = (*k*_3_/*K*_D3_) [S]/(1 + [S]/*K*_D_) yielded the *k*_3app_ values (*k*_3_/*K*_D3_) with their corresponding 95% confidence intervals. [S] in the above equation indicates lignosulfonate concentration referred to the basic phenylpropanoid unit, with molecular masses of 260 Da and 290 Da in the sulfonated softwood and hardwood lignins, respectively [26]. All measurements were at least quadruplicates.

### 2.9. Estimation of CI/RS, CI/CII and CII/RS Reduction Potentials

The reduction potentials (E°’) of the CI/RS and CII/RS pairs were calculated by stopped-flow spectrophotometry using the Nerst Equation (1) at equilibrium [44,45,46]:

ΔE°’ = (*RT*/*nF*) ln*K’* (1)

That correlates the difference of reduction potentials between enzyme and substrate with the equilibrium constant *K’*. *R* is equal to 8.31 J·K^−1^·mol^−1^, *T* is set to 298 K, *n* represents the number of electrons transferred in a single reaction step of the redox couple, and *F* (the Faraday constant) is 96,485 J·V^−1^·mol^−1^. *K’* represents the equilibrium constant, and is calculated as follows for the couples CI/RS (2) and CII/RS (3):

*K’* = ([H_2_O_2_][RS])/[CI] (2)

*K’* = ([Tyr^·^][RS])/([Tyr][CII]) (3)

The stopped-flow experiments, to quantify the different species at equilibrium, were as follows: (i) for E°’(CI/RS), 4 µM ApeLiP was mixed with different concentrations of H_2_O_2_ until equilibrium (i.e., no further progression of the reaction) was observed; and using their extinction coefficients at 410 nm (see below), the concentration of CI and RS were calculated; and (ii) for E°’(CII/RS) the RS enzyme (8 µM) containing 1 equiv of ferrocyanide was mixed with 2.5 equiv of H_2_O_2_ during 6 s to ensure CII formation; after that, different concentrations of tyrosine (which is oxidized by peroxidases to the tyrosyl radical, Tyr^·^) were added, and the concentrations of CII and RS were estimated with their *ε_410_* values. The concentrations at RS/CI and RS/CII equilibria were calculated using:

*A_410_* = *ε_410-RS_* [RS] l + *ε_410-CI_* [CI] l (4)

*A_410_* = *ε_410-RS_* [RS] l + *ε_410-CII_* [CII] l (5)

where *l* is the path length of the stopped-flow cuvette.

The extinction coefficient of CI (*ε_410-CI_* = 78,000 M^−1^·cm^−1^) was calculated after converting all RS to CI with 2.5 equiv of H_2_O_2_. The extinction coefficient of CII was considered the same as CI, since after mixing the enzyme with ferrocyanide and H_2_O_2_ no spectral changes were observed. All the experiments were carried out in 100 mM sodium tartrate, pH 3, using E°’(H_2_O_2_/H_2_O) = 1.56 V and E°’(Tyr^·^/Tyr) = 1.18 V, in the Nerst equation for the respective reduction potentials.

To infer the E°’ of CI/CII from the experimental values of the CI/RS and CII/RS couples, we took into account that the standard free energy of the reaction (G) and the E°’ are related according to the equation:

ΔG_r_’° = −n F E°’ (6)

with n = 2 electrons for the reduction of CI to RS, ΔG_r_’° equals to −2 F [E°’(CI/RS)] being the sum of the reaction free energy of one electron reductions (CI to CII, and CII to RS).

Therefore, the sum of reaction free energies ΔG_r_’°(CI/CII) + ΔG_r_’°(CII/RS) and the experimental determination of E°’(CII/RS) allows the determination of E°’(CI/CII).

### 2.10. Steady-State Treatment of Lignin

To evaluate lignin modification by ApeLiP, hardwood and softwood lignosulfonates (12 g·L^−1^) were treated in 50 mM phosphate, pH 5, at 25 °C for 24 h. The final concentration of enzyme was 1 µM (added in two equal doses at 0 and 12 h), and the final concentration of H_2_O_2_ was 10 mM, added continuously with a syringe pump. The reactions were carried out at pH 5, due to the higher 24 h stability of ApeLiP than at pH 3, where the stopped-flow measurements were carried out. Controls corresponded to the same reactions, but without enzyme.

### 2.11. 2D-NMR Analyses

The treated lignosulfonates and their controls were freeze-dried for NMR analyses. Spectra were recorded at 300 K on a Bruker Avance-III 500 MHz instrument equipped with a cryogenically cooled 5 mm TCI gradient probe with inverse geometry. The lignosulfonate samples (40 mg initial weight, before treatments) were dissolved in 0.6 mL of deuterated dimethylsulfoxide (DMSO-*d*_6_). The central peak of residual non-deuterated DMSO was used as internal reference (at δ_H_/δ_C_ 2.49/39.5 ppm), and the spectra were normalized to the same intensity of the DMSO signals, since the same final DMSO volume and initial amount of lignin was used in all the experiments.

The heteronuclear single-quantum correlation (HSQC) experiments used Bruker’s “hsqcetgpsisp.2” adiabatic pulse program with spectral widths from 0 to 10 ppm (5000 Hz) and from 0 to 165 ppm (20,625 Hz) for the ^1^H and ^13^C dimensions, respectively. The ^1^*J*_CH_ used was 145 Hz. HSQC spectra processing used typical matched Gaussian apodization in the ^1^H dimension and squared cosine-bell apodization in the ^13^C dimension. Signals were assigned by comparison with the literature [26,47,48,49,50,51,52].

In the aromatic region of the spectra, the H_2_-C_2_, H_5_-C_5_ and H_6_-C_6_ correlation signals were integrated to estimate the amount of lignin-units and the S/G ratio. In the aliphatic oxygenated region, the signals of methoxyls, and H_β_-C_β_ (or H_α_-C_α_) correlations in the side chains of sulfonated and non-sulfonated β-*O*-4′, phenylcoumaran and resinol substructures were integrated. The intensity corrections introduced by the adiabatic pulse program permits to refer the latter integrals to the previously obtained number of lignin units.

## 3. Results

### 3.1. Heterologous Expression, Protein Activation, and Purification of ApeLiP

A recent analysis of 52 Agaricomycetes genomes [29] revealed that the genome of *A. pediades* (https://mycocosm.jgi.doe.gov/Agrped1/Agrped1.home.html, accessed on 16 August 2021), a fungus belonging to the order Agaricales, contains a gene encoding a putative LiP enzyme (JGI protein ID# 705809, identified by the presence of a characteristic solvent exposed tryptophan residue and the absence of a Mn^2+^-oxidation site). To explore its functional and structural properties, ApeLiP was overexpressed in *E. coli*, where it accumulated as inclusion bodies.

**Figure 1:**
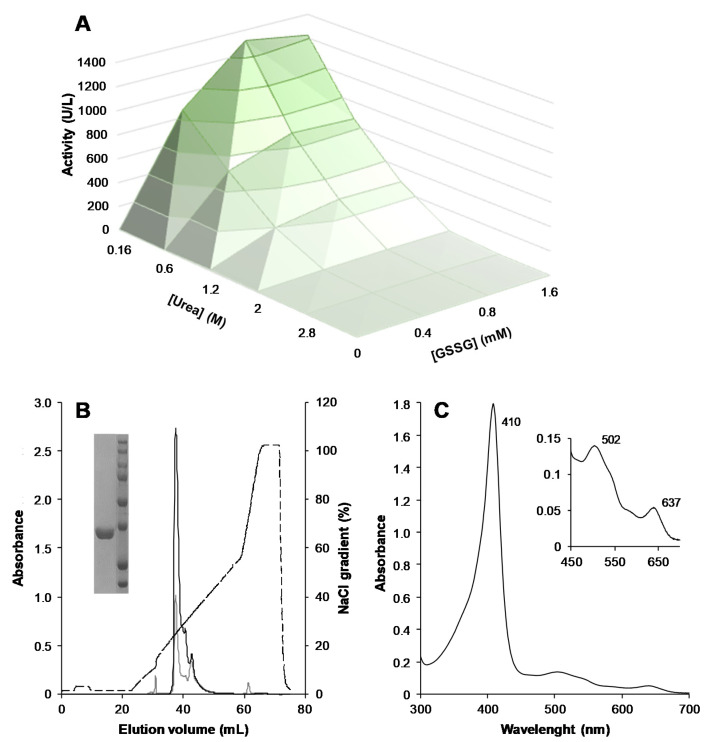
In vitro activation, purification, and electronic absorption spectrum of ApeLiP. (**A**) Screening of the optimal urea (0.16–2.80 M) and GSSG (0–1.6 mM) concentrations (by incubating 0.1 mg/mL protein in the presence of 0.1 mg/mL DTT, 0.02 mM EDTA, 5 mM CaCl_2_ and 15 μM hemin in 50 mM Tris-HCl, pH 9.0, at 4 °C during 24 h) as part of a multifactorial design. (**B**) Resource-Q chromatogram showing the elution profiles at 280 nm (gray line) and 410 nm (black line) and NaCl gradient (dashed line), with the inset showing SDS–PAGE of purified ApeLiP and molecular-mass markers (of 250, 150, 100, 75, 50, 37, 25 and 20 kDa, from top to bottom). (**C**) UV-visible spectrum of the enzyme at the resting state, with indication of the Soret band (410 nm) and CT1 (637 nm) and CT2 (502 nm) charge-transfer bands (inset).

Figure 1A shows the results of the protocol designed for optimizing the in vitro activation of recombinant ApeLiP, which yielded a maximum activity of ~1400 U/L (using ABTS as substrate) by exploring 672 folding conditions. After larger scale activation under the optimized conditions (i.e., folding mixture containing 0.16 M urea, 0.8 mM GSSG, 0.1 mM DTT, 20 µM EDTA, 5 mM CaCl_2,_ 15 μM hemin and 0.1 mg/mL protein in 50 mM Tris-HCl, pH 9, incubated at 4 °C for 24 h), ApeLiP was easily purified in a single anion-exchange chromatographic step (Figure 1B). The enzyme thus purified to electrophoretic homogeneity (Figure 1B, *inset*) showed a molecular mass in agreement with the theoretical mass calculated from the protein sequence (~34.7 kDa) and a Reinheitzahl value (A_410_/A_280_) of 2.6. Its UV-visible spectrum, shown in Figure 1C, is characteristic of a properly folded peroxidase with the heme cofactor correctly positioned in the active site. The Soret band with a maximum at 410 nm (close to that of LiPH2 from *P. chrysosporium*, identified at 409 nm [53]) and charge transfer bands (CT1 at 637 nm and CT2 at 502 nm) correspond to an enzyme in resting-state containing a high-spin ferric heme ready to be activated.

### 3.2. ApeLiP Crystallographic Studies

**Figure 2:**
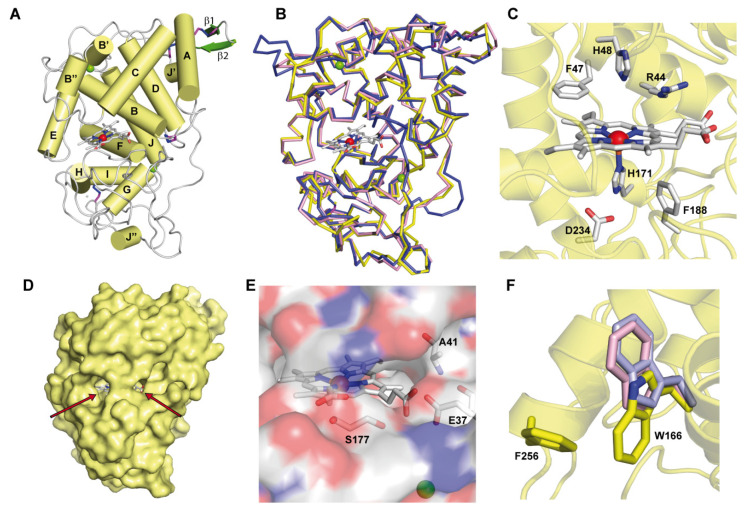
Crystal structure of ApeLiP. (**A**) Scheme of the overall structure showing 14 α-helices (yellow cylinders) and two small antiparallel β-strands (green), the heme cofactor (CPK sticks) with the iron center (red sphere), two structural calcium ions (green spheres), and four disulphide bonds (purple-blue sticks) (PDB entry 7OO5). (**B**) Structural alignment of the ApeLiP backbone (yellow) with the backbones of *P. chrysosporium* LiPA (blue) and *P. eryngii* VPL (magenta) (from PDB entries 1B82 and 2BOQ, respectively) (heme and structural calcium ions are also shown). (**C**) Heme cavity typical of class-II peroxidases including His171, Asp234 and Phe188 at the proximal side (below the heme plane); and His48, Arg44 and Phe47 at the distal side (above the heme plane) of ApeLiP. (**D**) Protein surface with indication (arrows) of two access channels to the heme cofactor. (**E**) Small access channel located on the heme propionates, with Ala41, Glu37 and Ser177 occupying the positions of the cation ligands in MnP and VP enzymes. (**F**) Superimposition of ApeLiP (yellow sticks), *P. chrysosporium* LiPA (blue), and *P. eryngii* VPL (magenta) catalytic tryptophans.

ApeLiP was successfully crystallized in the orthorhombic P2_1_2_1_2_1_ space group containing one molecule in the asymmetric unit. Its structure was determined by molecular replacement, and refined against the 1.85 Å diffraction data. The statistics of data collection, processing, and refinement are shown in Table 1. The ApeLiP refined structure gives the almost complete model. It consists of 331 residues (from Thr1 of the mature protein to Leu331, only lacking the C-terminal Arg332) and contains one heme molecule, two Ca^2+^ ions and two Zn^2+^ ions. The overall folding is mainly helical, with 14 α-helices, and one short antiparallel β-sheet (Figure 2A). Four disulfide bridges (Cys4-Cys16, Cys15-Cys279, Cys35-Cys116 and Cys245-Cys308) contribute to maintain the protein conformation together with the above Ca^2+^ ions, which are coordinated by Asp49, Gly63, Asp64 and Ser66 (Ca^2+^ at the heme distal side) and by Ser172, Asp196, Thr191, Leu194 and Asp189 (Ca^2+^ at the heme proximal side). Moreover, the presence of two structural zinc ions on the protein surface, from the crystallization medium, contributed to the folding stability.

The overall folding of ApeLiP can readily be superimposed with those of other ligninolytic peroxidases such as LiPA (isoenzyme H8, PDB 1B82) from *P. chrysosporium* and VPL (PDB 2BOQ) from *Pleurotus eryngii*, as revealed by the low r.m.s.d. values of 0.679 and 0.559 Å respectively (Figure 2B). As in these peroxidases, the heme cofactor is located in an internal pocket and its iron ion is coordinated by the Nε2 of one of two axial histidines (proximal His171 with a bond length of 2.27 Å, Figure 2C). The heme pocket also includes conserved Phe188 and Asp234 on the proximal side, along with Phe47, His48 and Arg44 on the distal side (Figure 2C), the two latter putatively involved in heme peroxidase activation by H_2_O_2_ [54]. 

Two channels give access to the distal side of the heme pocket (Figure 2D). The largest one corresponds to the channel enabling entrance of H_2_O_2_ for cofactor activation in all heme peroxidases, and also of low redox-potential dyes and phenols in different peroxidases [20,41,55]. The second channel is smaller and appears located directly on the heme propionates, occupying a position equivalent to that of the Mn^2+^-binding site in MnP and VP enzymes [22,29]. However, the Glu37/Ala41/Ser177 residues identified at the ApeLiP channel (Figure 2E) are not compatible with cation coordination.

**Figure 3:**
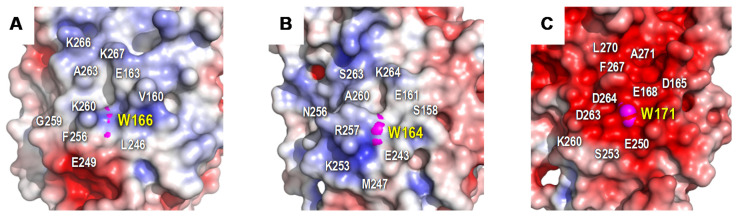
Electrostatic surface of the catalytic tryptophan environment in ApeLiP (**A**), *P. eryngii* VPL (**B**), and *P. chrysosporium* LiPA (**C**) calculated using the APBS-PDB2PQR software suite [56] with protonation states at pH 7.0 assigned by PROPKA [57]. Shown is the positioning of catalytic tryptophan (pink spheres) and close acidic and basic residues (together with other surrounding amino acids). Negative (red) and positive (blue) charges are indicated. PDB entries 7OO5 (**A**), 2BOQ (**B**), and 1B82 (**C**).

The crystal structure also presents a solvent exposed tryptophan (Trp166), as the one reported for oxidation of bulky and high redox-potential aromatic substrates including the lignin molecule [21,27]. Interestingly, its side chain in ApeLiP appears at a different orientation (turned by 180°) compared with previously crystallized LiPs and VPs (Figure 2F), probably due to hydrophobic interactions between the tryptophan indole group and the aromatic side chain of the adjacent Phe256. However, despite its different orientation, the indole group is sufficiently exposed in the surface of ApeLiP to putatively interact with and oxidize lignin. This oxidation would be modulated by the Trp166 environment that, in ApeLiP, is characterized by the presence of positively-charged (including Lys260, Lys266 and Lys267) together with negatively-charged amino acids, a feature also observed in VPs from Agaricales and Polyporales (Figure 3). In this way, only two acidic residues (Glu163 and Glu249) were found adjacent to ApeLiP Trp166, as also found in *P. eryngii* VPL (PDB 2BOQ) and in contrast with the five acidic residues present in the Trp171 environment of *P. chrysosporium* LiPA (PDB 1B82).

### 3.3. Temperature and pH Stability of Recombinant ApeLiP

**Figure 4:**
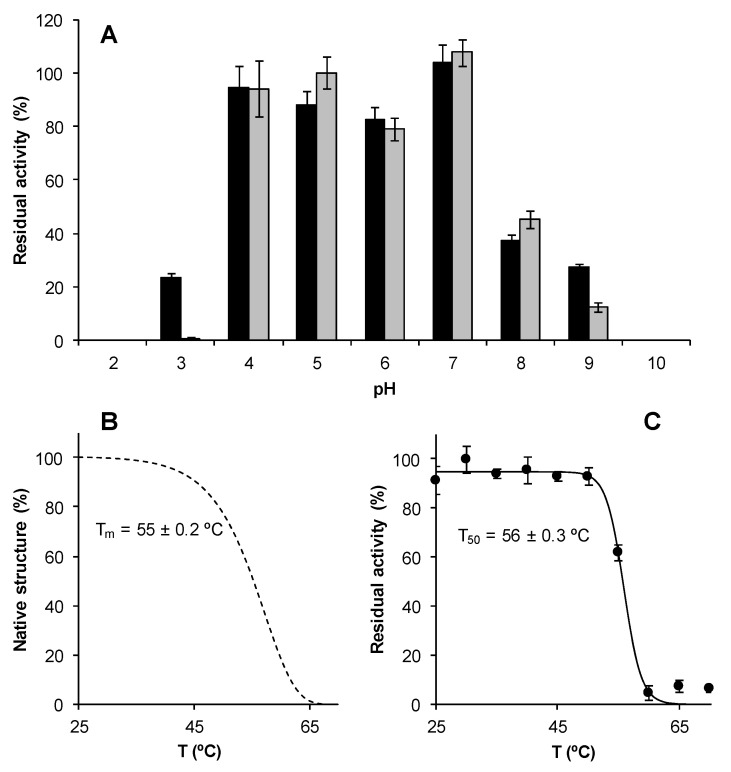
Effect of pH and temperature on ApeLiP stability. (**A**) pH stability as residual activities after 4 (black) and 24 (gray) hours incubation in 100 mM Britton–Robinson buffer (pH 2–10) at 25 °C. (**B**) Effect of temperature on protein denaturation measured by CD at 222 nm. (**C**) Thermal effect on ApeLiP activity after incubation in 10 mM sodium acetate, pH 5.5, for 10 min. T_m_ and T_50_ values are provided. Residual activities were measured with 1.25 mM ABTS and 0.1 mM H_2_O_2_ in 100 mM sodium tartrate, pH 3. Means and standard deviations are shown.

As shown in Figure 4A, ApeLiP is quite stable between pH 4.0 and 7.0, retaining ≥75% of its activity even after 24 h incubation. Out of this range, the stability strongly decreased, with full inactivation after 4 h at pH 2.0 and 10.0, as well as after 24 h at pH 3.0.

The effect of temperature on ApeLiP stability was analyzed by measuring both residual activity and CD melting profiles. As shown by the changes of secondary structure (from the ellipticity values at the 222 nm minimum in the 25–70 °C range), the protein underwent the first conformational changes at approximately 40 °C, being completely unfolded at ~65 °C. This resulted in a melting temperature (T_m_) of 55 ± 0.2 °C (Figure 4B) consistent with the activity T_50_ of 56 ± 0.3 °C (Figure 4C), which is similar to those observed in other ligninolytic peroxidases such as *P. ostreatus* VPs and MnPs [58]. 

### 3.4. Steady-State Kinetic Properties of ApeLiP

The catalytic properties of ApeLiP were analyzed using different phenolic and non-phenolic aromatic compounds. Reactions were carried out at the optimum pH for each substrate (with DMP oxidation being analyzed at both pH 3 and 8) (Figure S2A) under saturating H_2_O_2_ concentrations. Although the optimal pH for the oxidation of most substrates was pH 3.0 (pH 4.0 for VA), the stability abruptly falls when the enzyme is incubated for more than 4 h at this pH value, a fact that has to be taken into account for setting up reaction conditions.

As shown in Table 2, ApeLiP is able to transform simple high redox-potential aromatics. Thus, the azo dye RB5 and the non-phenolic lignin model compound VA were oxidized by the enzyme, although the latter was oxidized with a much lower catalytic efficiency. As expected from the structural analysis provided above, no activity towards Mn^2+^ was detected (as found in MnPs and VPs). The low redox-potential dye ABTS was efficiently oxidized by ApeLiP. This reaction fits a biphasic kinetic curve (Figure S3) revealing the involvement of two catalytic sites for this substrate, characterized by high (*k*_cat_*/K*_M_ = 5130 ± 460 s^−1^·mM^−1^) and low (*k*_cat_*/K*_M_ = 258 ± 25 s^−1^·mM^−1^) efficiencies (Table 2). By contrast, oxidation of DMP, another low redox-potential compound that represents the minor phenolic moiety of lignin, shows a Michaelis–Menten behavior with a catalytic efficiency (*k*_cat_*/K*_M_ = 1910 ± 310 s^−1^·mM^−1^) in the order of the ABTS high-efficiency site, and inhibition at higher substrate concentrations (Figure S3).

As a closer approach to study the ligninolytic capabilities of ApeLiP, GGE and VGE dimers were used as phenolic and non-phenolic substrates, respectively. ApeLiP was able to oxidize both substrates (Figure S4), requiring higher enzyme (8-fold) dosage to observe VGE oxidation. The optimal pH was different for the two non-phenolic lignin model compounds assayed, being pH 4 for the oxidation of VA (model monomer) and pH 3 for the oxidation of VGE (model dimer) (Figure S2).

### 3.5. Identification of Trp166 as a Catalytic Residue

To confirm the putative catalytic role of the solvent-exposed Trp166 in ApeLiP, the W166A variant was obtained by PCR mutagenesis, expressed, purified, and its steady-state kinetic constants compared to those of the wild-type enzyme. As shown in Table 2, substitution of this tryptophan by alanine led to drastic changes in the ApeLiP catalytic performance, losing its capability of oxidizing high redox-potential substrates such as RB5 and VA. Similarly, the high efficiency oxidation of ABTS was completely suppressed, and strong substrate inhibition was observed at the low efficiency site in the W166A variant (*k_i_* was not calculated since data could not be fitted to the inhibition equation).

This mutation also caused the loss of activity on DMP below pH 7 and shifted the optimal pH from 3 to 8 (Figure S2B). The DMP kinetics of ApeLiP and its W166A variant at pH 8 showed strong substrate inhibition, and lower catalytic efficiency values than that of the wild-type enzyme at pH 3, suggesting that DMP could be oxidized in a low efficiency catalytic site at pH 8 and in contact with Trp166 at pH 3. All these results revealed that the exposed Trp166 is a key residue for ApeLiP activity towards both high and low redox-potential aromatic substrates.

### 3.6. Catalytic Cycle and ApeLiP Reduction Potentials

**Figure 5:**
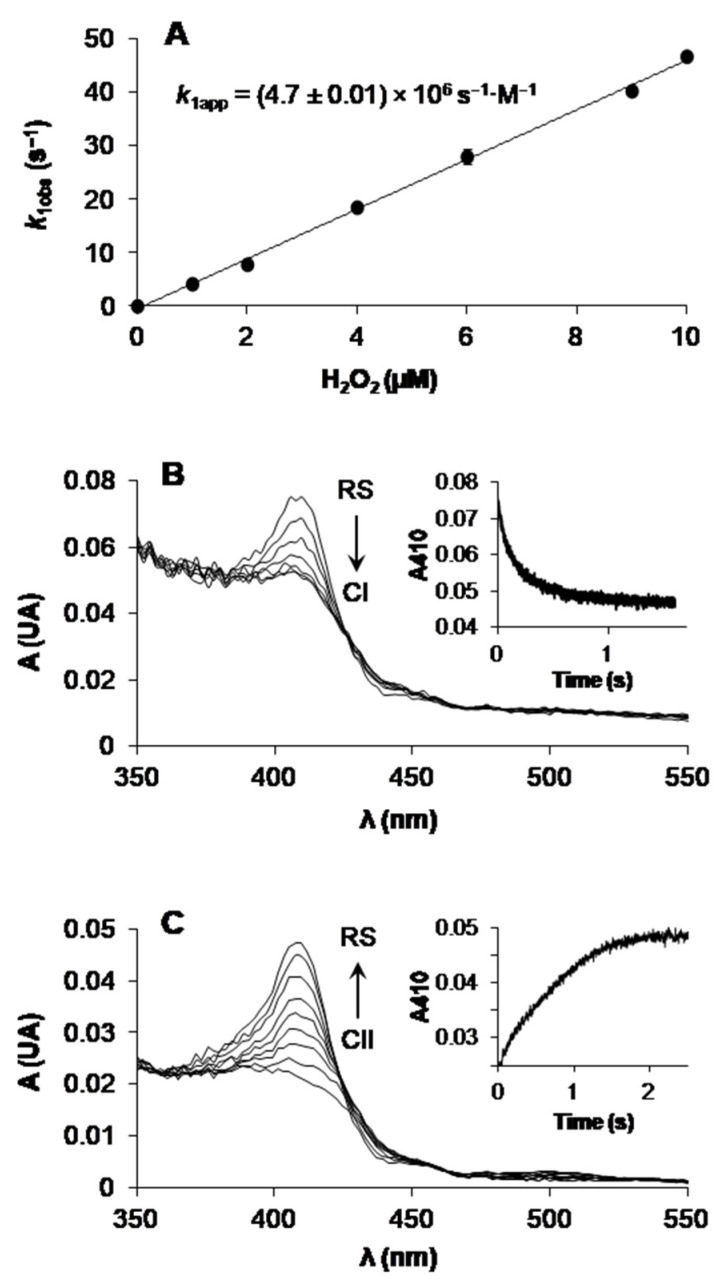
Stopped-flow spectrophotometric analysis of ApeLiP. (**A**) CI formation by H_2_O_2_ followed at 397 nm (means and 95% confidence limits are shown). (**B**) Spectral changes upon mixing RS enzyme with H_2_O_2_ to attain the RS/CI equilibrium. (**C**) Spectral changes upon mixing CII enzyme (formed by adding 2.5 equiv of H_2_O_2_ and 1 equiv of ferrocyanide) with tyrosine to attain the CII/RS equilibrium. The insets show time traces at the Soret band (410 nm) to attain equilibrium concentrations. All reactions were carried out at pH 3 and 25 °C. See Tables S1 and S2 for estimation of the E°’(CI/RS) and E°’(CII/RS) values, respectively.

The catalytic cycle of ligninolytic peroxidases (Figure S1) starts with a two-electron oxidation of the RS enzyme by H_2_O_2_, forming CI. This intermediate is then reduced back to the RS enzyme via CII by two one-electron substrate oxidations in direct contact with the heme cofactor or at the solvent-exposed tryptophan. CI formation in ApeLiP was followed by stopped-flow spectrophotometry at 397 nm. A linear dependence on H_2_O_2_ concentration was observed yielding an apparent second-order rate constant (*k*_1app_) (Figure 5A). To explore the formation of ApeLiP CII, CI was mixed with different concentrations of ferrocyanide to generate CII. Unfortunately, no spectral changes were observed suggesting that CI and CII are not spectrophotometrically distinguishable.

The reduction potential of CI to RS (E°’[CI/RS]) could be measured at the equilibrium during the RS reaction with different H_2_O_2_ concentrations (the time-course of one of these oxidation reactions is shown in Figure 5B). Similarly, the reduction potential of the CII-like species mentioned above (generated with H_2_O_2_ and ferrocyanide) to RS (E°’[CII/RS]) was estimated at the equilibrium with different concentrations of tyrosine as reducing substrate (the time-course of one of these reduction reactions is shown in Figure 5C). Using the Nerst equation at the different redox equilibria, the average values of E°’(CI/RS) = 1.367 ± 0.004 V (Table S1) and E°’(CII/RS) = 1.281 ± 0.012 V (Table S2) were obtained. Finally, we were able to infer the remaining reduction potential of the catalytic cycle, E°’(CI/CII), from the experimentally determined values of the CI–RS and CII/RS pairs, as described in Materials and Methods, and a value of 1.453 ± 0.004 V was obtained.

### 3.7. Lignin Oxidation by ApeLiP: Transient-State Kinetic Data and NMR Analyses

**Figure 6:**
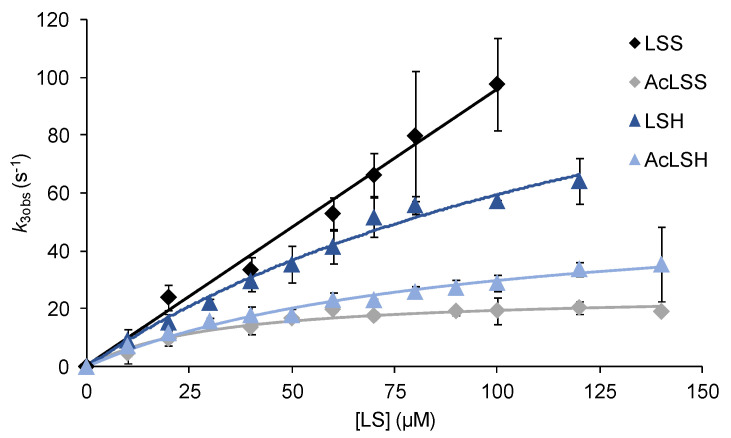
Kinetics of ApeLiP CII reduction to RS by native (black and dark blue) and acetylated (gray and light blue) softwood (LSS) and hardwood (LSH) lignosulfonates. Stopped-flow reactions were carried out in 100 mM sodium tartrate, pH 3, at 25 °C. Lignosulfonate concentrations refer to the basic phenylpropanoid unit. Means and 95% confidence limits are shown.

To evaluate the putative contribution of ApeLiP to the ligninolytic capabilities of *A. pediades*, we first analyzed by stopped-flow rapid spectrophotometry the ability of the enzyme to extract electrons directly from lignin, focusing on the rate-limiting step. The kinetics for the resulting reduction of ApeLiP CII to its RS at pH 3 (the optimum previously determined for the oxidation of lignin model dimers) by the native and acetylated (with the hydroxyl groups of their minor phenolic moiety blocked by ester linkages) softwood (*P. abies*) and hardwood (*E. grandis*) lignosulfonates is compared in Figure 6.

Table 3 shows the transient-state kinetic parameters for CII reduction in the reactions analyzed here, together with those previously reported for other ligninolytic peroxidases. The ApeLiP results confirm better oxidation of native (non-acetylated) lignosulfonates, with similar efficiency (*k*_3app_) values for softwood (926 ± 47 s^−1^·mM^−1^) and hardwood (957 ± 270 s^−1^·mM^−1^) lignins. Compared with other enzymes, ApeLiP is more efficient than *P. eryngii* VP and *P. chrysosporium* LiP oxidizing both native and acetylated lignosulfonates (with the exception of native hardwood lignosulfonate being slightly better oxidized by *P. eryngii* VP). ApeLiP was also able to extract electrons from non-phenolic acetylated lignosulfonates, being more efficient oxidizing the acetylated softwood lignin. The acetylation of lignin lowered the electron transfer rates, indicating that the phenolic units are easier to be oxidized by the enzyme. The action of the W166A variant was also tested and no significant CII reduction was observed by any of the lignosulfonate samples, confirming the key role of Trp166 oxidizing both phenolic and non-phenolic lignins.

**Figure 7:**
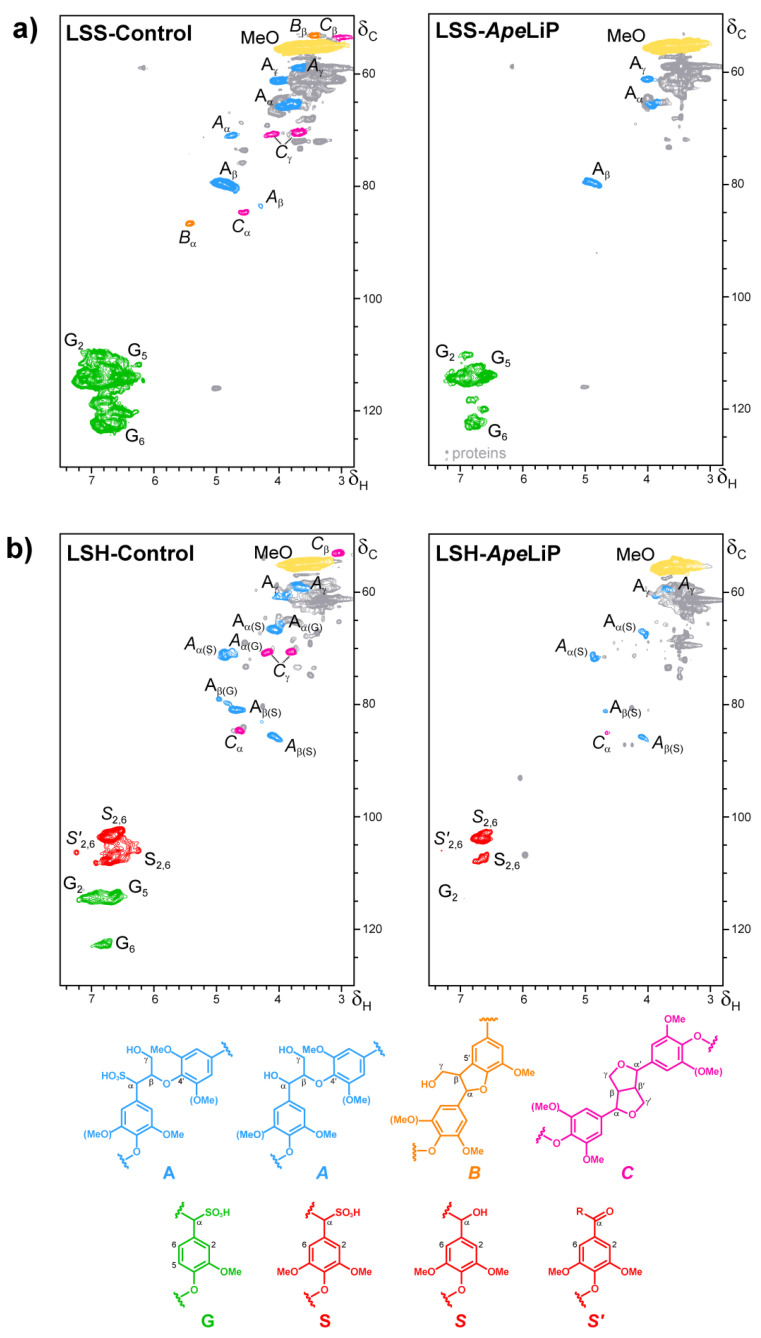
Normalized HSQC NMR spectra of softwood (*P. abies*, (**a**) and hardwood (*E. grandis*, (**b**) lignosulfonates treated for 24 h with ApeLiP (right) and non-treated (left), with the formulae of the main substructures indicated below. Signals correspond to ^1^H–^13^C correlations at the different positions of Cα-sulfonated guaiacyl units (**G**); Cα-sulfonated, nonsulfonated, and Cα-oxidized syringyl units (**S**, ***S***, and ***S*′**, respectively); Cα-sulfonated and nonsulfonated β-O-4′ substructures (**A** and ***A***, respectively), phenylcoumaran (**B**) and resinol (**C**) substructures; and methoxyls (**MeO**). Spectra correspond to the same initial amount of lignosulfonate (40 mg) in the control and enzyme-treated samples, and their normalized intensities are referred to the signal (not shown) of residual non-deuterated DMSO.

Additionally, to detect changes in lignin structure by ApeLiP, lignosulfonates were analyzed by HSQC 2D-NMR spectroscopy after 24 h reactions. The signals of the main aromatic units and side-chain inter-unit linkages in softwood and hardwood lignosulfonates were well resolved (Figure 7), including Cα-sulfonated guaiacyl units (G) and Cα-sulfonated, nonsulfonated, and Cα-oxidized syringyl units (S, *S* and *S’*, respectively); together with Cα-sulfonated and nonsulfonated β-O-4′ substructures (A and *A*, respectively) and less abundant phenylcoumaran (B) and resinol (C) substructures. The intensities of the above signals in the treated samples and controls were normalized to the residual (non-deuterated) DMSO signal used as an internal standard. A semiquantitative analysis of the aromatic signals (Table S3 part-a), referred to the control without enzyme, enabled to calculate the lignin decay and the modification of S/G ratio and side-chain linkages. The action of ApeLiP caused 71% decrease of aromatic signals in the softwood lignosulfonate and even higher decreases (92% decrease of G units, and 82% of S units) in the hardwood lignosulfonate (its S/G ratio passing from 2.2 to 4.7). However, the side-chain linkages per aromatic unit were not strongly modified (from 38 to 31% in softwood lignosulfonate and from 35 to 32% in hardwood lignosulfonate) and the relative abundances of the different substructures (A, B, and C) were basically maintained (Table S3 part-b).

## 4. Discussion

### 4.1. Lignin Peroxidase in Agaricales Genomes

Fungal degradation of lignin has been traditionally studied in Polyporales, where most wood-rotting fungi are included [60]. Extensive genomic analysis of these organisms and biochemical characterization of their enzymes have highlighted the key role of high redox-potential fungal peroxidases in lignin degradation [9]. Among them, LiPs stand out for their capacity to oxidize the major non-phenolic moiety of the lignin macromolecule [16]. Conversely, lignin degradation by Agaricales has been barely studied, in spite of being the largest basidiomycetes order with around 13,000 described species [61], and representing a potential source of new lignocellulolytic enzymes given their growth on a variety of lignocellulosic materials. A recent thorough study of 52 Agaricomycetes genomes has revealed the high presence of putative ligninolytic peroxidases in fungi belonging to the order Agaricales [29]. These include the first LiP outside the order Polyporales, identified in the genome of the mushroom *A. pediades* (ApeLiP) as a case of parallel and convergent evolution of LiPs between Agaricales and Polyporales, whose main similarities and differences are discussed below.

### 4.2. ApeLiP Activation and LRET Oxidation of Lignin Models

The crystal structure of ApeLiP maintains the heme cavity architecture found in other ligninolytic peroxidases [62]. The cofactor is buried in the structure and sandwiched by proximal (F) and distal (B) helices, which contain conserved residues involved in the heterolytic cleavage of H_2_O_2_ to form CI (at the distal side) and in the modulation of the redox-potential of the enzyme (with a proximal histidine acting as fifth ligand of the heme iron). Therefore, it was expected to observe a similar enzyme activation by H_2_O_2_. In fact, its apparent second-order rate constant for CI formation, (4.7 ± 0.01) × 10^6^ s^−1^·M^−1^, is in the same range of other ligninolytic peroxidases such as *P. eryngii* VP [43], *P. chrysosporium* MnP [42] and *Trametes cervina* LiP [63], although this rate was one order of magnitude higher than reported for *P. chrysosporium* LiPA (isoenzyme H8) [64]. These differences in the reaction rate with H_2_O_2_ could affect the oxidative stability of peroxidases, as confirmed by a VP variant at the heme distal side showing slower activation by H_2_O_2_ and enhanced oxidative stability [65].

A putative oxidation site was identified at the protein surface including a solvent-exposed tryptophan. This tryptophan (Trp166 in ApeLiP), conserved in most LiPs and VPs, would be involved in the oxidation of high redox-potential and bulky molecules, including lignin [21,26,27], by long-range electron transfer (LRET) from the protein surface to the activated heme cofactor [66]. To evaluate this hypothesis, the reactions catalyzed by the wild-type enzyme and its tryptophan-less W166A variant were kinetically characterized. ApeLiP was able to oxidize the non-phenolic lignin model dimer VGE (and also VA). Moreover, this enzyme oxidizes high redox-potential dyes such as RB5 without the requirement of redox mediators, a feature only observed in VPs [43,67] and ancestral LiPs [23,44,68], but not in *P. chrysosporium* LiPA and *Bjerkandera adusta* LiP2 that require VA for efficient oxidation of different aromatic compounds and dyes [69,70,71]. As no activity was observed when the W166A mutation was present, the importance of this residue in degradation of non-phenolic lignin and other recalcitrant molecules by ApeLiP stands clear.

Moreover, phenolic lignin model compounds (GGE and DMP) and low-redox-potential dyes (ABTS) are oxidized with higher catalytic efficiencies than observed for other LiPs [19] and VPs [41]. Besides, biphasic kinetics were observed for ABTS oxidation, indicating the presence of high and low efficiency oxidation sites [41]. Disappearance of high-efficiency oxidation of phenolic compounds when Trp166 was removed suggests that this residue would also play a key role in the oxidation of phenolic lignin by ApeLiP.

The protein environment around the catalytic tryptophan can significantly affect substrate recognition and/or oxidation by LiPs and VPs. An electronegative environment would stabilize the VA cation radical to act as an enzyme-bound redox mediator [72] at the same time that it can displace the VA oxidation reaction toward the oxidized forms. Moreover, acidic residues would lower the local pH, providing a higher redox-potential to the tryptophanyl radical [73]. The less acidic environment of Trp166 could explain the lower oxidation power of ApeLiP on VA, with its activity and substrate recognition properties closer to those of VPs than of LiPs.

### 4.3. Heme-Channel Oxidation Site

As reported for plant and fungal generic peroxidases and for *P. eryngii* VP, phenolic and other low redox-potential substrates can be oxidized directly by the heme through the channel that gives access to H_2_O_2_ [41,55]. However, the narrowness of this channel in most LiPs impedes these aromatic substrates to directly interact with the cofactor [62]. The size and shape similarities between the ApeLiP and VP heme channels may explain the kinetic identification of a low-efficiency site for ABTS oxidation in the former enzyme, which remained when Trp166 was removed. Moreover, although the catalytic tryptophan was responsible for high-efficiency oxidation of DMP at pH 3, its optimal oxidation by the W166A variant was at pH 8, suggesting that this substrate may be oxidized by the heme cofactor under more basic conditions.

The ApeLiP ability to oxidize aromatic substrates (VGE included) at basic pH is a unique characteristic among the best known ligninolytic peroxidases, which are inactivated under these conditions due to Ca^2+^ loss and heme pocket collapse with hexacoordination of the heme iron [74,75,76,77]. Although the exact structural basis for this “basic peroxidase” activity remains to be identified, it seems related to direct substrate oxidation at the heme channel, in agreement with DMP oxidation results by the Trp-less ApeLiP variant. Catalytic activity at basic pH has been built in a VP by directed evolution, and the oxidation of low-redox potential substrates in direct contact with the heme cofactor was related with the stabilization of its heme pocket at alkaline pH [78,79]. This suggests a higher stability of the heme and its environment in ApeLiP at basic pH compared to other ligninolytic peroxidases.

### 4.4. Intriguing Catalytic Cycle and Lignin Decay Abilities

The stopped-flow spectrophotometric inspection of the ApeLiP catalytic cycle also showed differences with respect to LiPs from Polyporales. The recently studied evolution of reduction potential in these ligninolytic peroxidases shows an increase through time for all the catalytic pairs, culminating in the highest E°’ of extant LiPs [44]. However, ApeLiP showed comparatively low E°’ values. Those of its CI/RS, CI/CII and CII/RS pairs (Figure S1) follow the general tendency in other peroxidases, being E°’(CII/RS) the lowest (as corresponds to the rate-limiting step) and E°’(CI/CII) the highest (explaining the instability of CI). However, all the values for ApeLiP are up to 70 mV lower than the reduction potentials observed for LiPs of Polyporales. Additionally, the catalytic cycle of ApeLiP is intriguing. We were unable to discriminate CI from CII using stopped-flow rapid spectrophotometry after ferrocyanide addition as classically used to generate peroxidase CII [80,81]. However, the putative intermediate forms the RS enzyme upon mixing with reducing substrate (either tyrosine or lignosulfonates) behaving as the typical CII of other ligninolytic peroxidases [44,59]. 

Despite its lower reduction potential, lignin oxidation by ApeLiP was not compromised. Transient-state kinetics with lignosulfonate substrates confirmed that ApeLiP can oxidize lignin from different origins with similar efficiency (*k*_3app_) values, unlike previously reported for *P. chrysosporium* LiPA [59]. Moreover, ApeLiP was still active after blocking the phenolic groups by acetylation of lignosulfonates, proving its capability to oxidize the major non-phenolic lignin moiety. Comparing the reduction of CII to RS enzyme, the rate-limiting step in lignin oxidation, the observed transient-state rate constants for ApeLiP were similar or slightly higher than those reported for *P. eryngii* VPL [27] and *P. chrysosporium* LiPA [59]. Moreover, in agreement with results using model compounds, we barely detected any activity on lignosulfonates with the W166A variant, definitively confirming the role of Trp166 oxidizing both the phenolic and non-phenolic units of lignin. Interestingly, the efficiency of ApeLiP oxidizing the acetylated softwood lignosulfonate was higher than those of *P. chrysosporium* LiP and *P. eryngii* VP acting on the same native lignosulfonate, a result that reveals Agaricales LiP as the best biocatalyst oxidizing softwood lignin described to date.

Finally, using 2D-NMR, we observed a considerable decay of softwood lignosulfonate, and even more of hardwood lignosulfonate, by ApeLiP, resulting in strong decreases in the intensities of the lignin aromatic signals. In contrast, only slight modification in the proportion of lignin substructures with different inter-unit linkages was observed. Interestingly, ApeLiP causes stronger modification of G lignin than *P. chrysosporium* LiPA in steady-state treatments under similar reaction conditions [59]. The above was not only evidenced by stronger lignin modification when using softwood lignosulfonate as ApeLiP substrate, but also by the preferential removal of G units during the treatment of hardwood lignin. The results here obtained suggest that ApeLiP could play a role in plant biomass degradation by *A. pediades*, and that LiPs with relevant ligninolytic capabilities, resulting from an evolutionary pathway different from those of Polyporales LiPs [29], exist in Agaricales.

## 5. Conclusions

Mushroom ligninolytic machineries have been understudied despite being potential sources of oxidoreductases with biotechnological potential. Here, we characterized for the first time a LiP enzyme identified in the genome of *A. pediades*, a saprotrophic basidiomycete of the order Agaricales. Its crystal structure, kinetic characterization, and the thorough study of its ligninolytic capability shows that this LiP is able to oxidize both phenolic and non-phenolic lignin model compounds and real lignin, the latter in similar or higher extent than other well-known ligninolytic peroxidases. Therefore, not only wood-rotting Polyporales, but also Agaricales mushrooms, have enzymes with high relevance for both carbon recycling in nature and biotechnological modification of lignin.

## Figures and Tables

**Table 1:** Crystallographic data collection and refinement statistics of ApeLiP.

Data Collection		Refinement	
Resolution range (Å)	74.95–1.85 (1.95–1.85)	Resolution (Å)	55.56–1.85
Space group	P 2_1_ 2_1_ 2_1_	Working reflections	26,256 (2733)
Unit cell (Å)	a = 48.13, b = 74.95, c = 82.77	Reflections test set	1270 (140)
Total reflections	164,812 (23,933)	R-work (%)	22.6 (40.8)
Unique reflections	26,316 (3760)	R-free (%)	26.8 (42.1)
Multiplicity	6.3 (6.4)	Number of non-H atoms	
Completeness (%)	99.8 (100.0)	- protein	2413
Mean I/sigma (I)	15.5 (3.0)	- heme group	43
Wilson B-factor (Å^2^)	35.8	- ions	4
R_merge_	0.051 (0.483)	- solvent	83
R_meas_	0.061 (0.575)	Average B-factor (Å^2^)	
R_pim_	0.033 (0.309)	- protein	48.02
CC_1/2_	0.99 (0.93)	- heme group	33.78
		- ions	43.51
		- solvent	45.56
		rmsd bond lengths (Å)	0.012
		rmsd angles (°)	1.67
		Ramachandran statistics	
		- Preferred (%)	92.71
		- Allowed (%)	5.78
		- Outliers (%)	1.52
		PDB code	7OO5

Statistics for the highest-resolution shell are shown in parentheses.

**Table 2:** Steady-state kinetic parameters—*K*_M_ (µM), *k*_cat_ (s^−1^), *k*_cat_/*K*_M_ (s^−1^·mM^−1^), and *k*_i_ (µM)—for H_2_O_2_, ABTS, DMP, VA, and RB5 reactions of ApeLiP and its W166A variant.

			ABTS ^a^		DMP			
		H_2_O_2_	Low Efficiency	High Efficiency	pH 3	pH8	VA	RB5
ApeLiP	*K*_M_	33 ± 4.4	693 ± 63	8.1 ± 0.6 ^b^	37 ± 5.6	1430 ± 440	(29.6 ± 4.6) × 10^3^	14 ± 1.4 ^b^
	*k*_cat_	100 ± 4.8	179 ± 5	42 ± 1.8	68.6 ± 2.8	5.7 ± 0.7	4.3 ± 0.3	21 ± 1.5
	*k*_cat_/*K*_M_	3070 ± 440	258 ± 25	5130 ± 460	1910 ± 310	4.0 ± 1.3	0.15 ± 0.03	1540 ± 115
	*k*_i_	(2.1 ± 0.5) × 10^3^	-	-	(11 ± 2.2) × 10^3^	nd ^d^	(406 ± 92) × 10^3^	-
W166A	*K*_M_	136 ± 12	1150 ± 190 ^c^	-	-	49 ± 8.2	-	-
	*k*_cat_	126 ± 4.7	117 ± 10 ^c^	-	-	6.1 ± 0.27	-	-
	*k*_cat_/*K*_M_	929 ± 87	102 ± 19 ^c^	-	-	125 ± 22	-	-
	*k*_i_	(3.3 ± 0.3) × 10^3^	nd ^c^	-	-	nd ^d^	-	-

Reactions in 100 mM sodium tartrate or Tris-HCl buffer at 25 °C under H_2_O_2_ saturation (0.4 mM for ApeLiP and 1 mM for W166A). H_2_O_2_ reduction (estimated with 5 mM ABTS as reducing substrate) and ABTS and RB5 oxidation were measured at pH 3.0, DMP oxidation at pH 3.0 and 8.0, and VA oxidation at pH 4.0. Unless otherwise stated, Michaelis–Menten or inhibition equations were used. ^a^ Biphasic kinetics yielding two sets of constants. ^b^ Data from high-efficiency oxidation of ABTS and RB5 adjusted to Hill equation (n = 1.62 ± 0.12 and 2.11 ± 0.30, respectively; *K*_0.5_ considered equivalent to *K*_M_). ^c,d^ Substrate/product inhibition with parameters estimated from Hill (n = 1.62 ± 0.28) and Michaelis–Menten equations, respectively, as data did not fit to the inhibition equation. nd, not determined. -, no activity. Means and standard deviations are shown.

**Table 3:** Transient-state kinetic parameters—*K*_D3_ (µM), *k*_3_ (s^−1^), and *k*_3app_ (s^−1^·mM^−1^) —for rate-limiting reduction of CII of ApeLiP, its W166A variant, *P. eryngii* VPL, and *P. chrysosporium* LiPA (isoenzyme H8) by native and acetylated softwood and hardwood lignosulfonates ^a^.

		Softwood Lignin		Hardwood Lignin	
		Native	Acetylated	Native	Acetylated
ApeLiP	*K*_D3_	ns ^b^	31 ± 6.9	156 ± 25	89 ± 14
	*k*_3_	ns	26 ± 1.7	163 ± 38	56 ± 4.6
	*k*_3app_	926 ± 47	830 ± 195	957 ± 270	630 ± 111
W166A	*K*_D3_	-	-	-	-
	*k*_3_	-	-	-	-
	*k*_3app_	0	0	0	0
*P. eryngii* VPL ^d^	*K*_D3_	143 ± 19	24 ± 1.9	14 ± 1	21 ± 2.5
	*k*_3_	48 ± 2	14 ± 0.4	14 ± 2	12 ± 0.5
	*k*_3app_	340 ± 30	599 ± 31	990 ± 80	592 ± 52
*P. chrysosporium* LiPA ^e^	*K*_D3_	95 ± 26	na ^c^	19 ± 2	na
	*k*_3_	25 ± 4	na	14 ± 0	na
	*k*_3app_	263 ± 83	na	764 ± 86	na

^a^ Reactions in 0.1 M sodium tartrate, pH 3, at 25 °C. ^b^ ns, non-saturation kinetics. ^c^ na, not available. ^d^ From [27]. ^e^ From [59]. Means and 95% confidence limits.

## Data Availability

All data underlying this article are available in the main publication and in its Supplementary Material online. Any additional information can be obtained on request to the corresponding authors.

## Supplementary Materials

The following are available online at https://www.mdpi.com/article/10.3390/antiox10091446/s1, Table S1: parameters of the redox equilibrium and calculated E°’ of the CI/RS pair of ApeLiP, Table S2: parameters of the redox equilibrium and calculated E°’ of the CII/RS pair of ApeLiP, Table S3: semiquantitative HSQC-NMR analysis of softwood and hardwood lignosulfonates after 24 h treatment with ApeLiP, Figure S1: catalytic cycle of ligninolytic peroxidases and E°’ of the ApeLiP CI/RS, CI/CII and CII/RS pairs, Figure S2: optimal pH for the oxidation of high and low redox-potential substrates by ApeLiP and its W166A variant, Figure S3: kinetic curves for substrate oxidation by ApeLiP and its W166A variant, Figure S4: time courses of ApeLiP reactions with GGE and VGE lignin model dimers.
