# Supplementary material for: Agaricales Mushroom Lignin Peroxidase: From Structure–Function to Degradative Capabilities
Source: Antioxidants (Basel). 2021 Sep 12;10(9):1446. doi: 10.3390/antiox10091446 (PMC8472802; doi:10.3390/antiox10091446)
Supplement: Supplementary file 1 [file antioxidants-10-01446-s001.zip › antioxidants-1365360-supplementary.pdf]

antioxidants

MDPI

Supplementary Materials: Agaricales Mushroom Lignin Peroxidase: From Structure–Function to Degradative Capabilities

María Isabel Sánchez-Ruiz [1,†], Iván Ayuso-Fernández [1,†,‡], Jorge Rencoret [2], Andrés Manuel González-Ramírez [1,§], Dolores Linde [1], Irene Davó-Siguero [1], Antonio Romero [1], Ana Gutiérrez [2], Angel T. Martínez[1,*] and Francisco Javier Ruiz-Dueñas [1,*]

[1] Centro de Investigaciones Biológicas "Margarita Salas" (CIB), Consejo Superior de Investigaciones Científicas (CSIC), 28040 Madrid, Spain; marisasr@cib.csic.es (M.I.S.-R.); ivan.ayuso-fernandez@nmbu.no (I.A.-F.); amgonram@gmail.com (A.M.G.-R.); lolalinde@cib.csic.es (D.L.); ireneids3@gmail.com (I.D.-S.); romero@cib.csic.es (A.R.)
[2] Instituto de Recursos Naturales y Agrobiología de Sevilla (IRNAS), Consejo Superior de Investigaciones Científicas (CSIC), 41012 Seville, Spain; jrencoret@irnase.csic.es (J.R.); anagu@irnase.csic.es (A.G.)
* Correspondence: atmartinez@cib.csic.es (A.T.M.); fjruiz@cib.csic.es (F.J.R.-D.); Tel.: +34-(91)-8373112 (ext. 4407/4445) (A.T.M.); Tel.: +34-(91)-8373112 (ext. 4253/4258) (F.J.R.-D.)
† These two authors contributed equally to this work
‡ Current address: Norwegian University of Life Sciences (NMBU), 1430 Ås, Norway
§ Current address: Institute for Biocomputation and Physics of Complex Systems, 50013 Zaragoza, Spain

This Supplementary Information includes: Parameters of the redox equilibrium and calculated E°' of the CI/RS pair of ApeLiP (**Table S1**), parameters of the redox equilibrium and calculated E°' of the CII/RS pair of ApeLiP (**Table S2**), semiquantitative HSQC-NMR analysis of softwood and hardwood lignosulfonates after 24-h treatment with ApeLiP (**Table S3**), catalytic cycle of ligninolytic peroxidases and E°' of the ApeLiP CI/RS, CI/CII and CII/RS pairs **(Figure S1)**, optimal pH for the oxidation of high and low redox-potential substrates by ApeLiP and its W166A variant (**Figure S2**), kinetic curves for substrate oxidation by ApeLiP and its W166A variant (**Figure S3)**, time courses of ApeLiP reactions with GGE and VGE lignin model dimers (**Figure S4**).

Table S1. Parameters of the redox equilibrium and calculated E°' of the CI/RS pair of ApeLiP as a function of the initial concentration of H_2O_2.[a]

Initial H_2O_2 (µM)	Equilibrium concentrations (µM)			E°' (V)
	RS-ApeLiP	CII-ApeLiP	H_2O_2	
1	0.74	2.36	0.26	1.380
2	1.55	1.55	0.45	1.372
2.5	2.17	0.93	0.33	1.358
3	2.56	0.54	0.44	1.352
		Mean and 95% confidence interval:		1.365 ± 0.012

[a] Reactions were performed in 100 mM sodium tartrate, pH 3.0.

Table S2. Parameters of the redox equilibrium and calculated E°' of the CII/RS pair of ApeLiP as a function of the initial concentration of Tyr.[a]

Initial Tyr	Equilibrium concentrations (µM)				E°' (V)
(µM)	RS-ApeLiP	CII-ApeLiP	Tyr	Tyr·	
5	0.26	1.67	4.74	0.26	1.299
10	0.49	1.44	9.51	0.49	1.280
20	0.74	1.19	19.26	0.74	1.272
25	0.79	1.14	24.21	0.79	1.273
		Mean and 95% confidence interval:			1.281 ± 0.012

[a] Reactions were performed in 100 mM sodium tartrate, pH 3.0.

Table S3. Semiquantitative HSQC-NMR analysis of softwood (*P. abies*) and hardwood (*E. grandis*) lignosulfonates after 24-h treatment with ApeLiP including: **a)** Lignin composition (G and S units) and decay (parentheses); and **b)** Side-chain linkages as relative percentages and referred to 100 lignin units (parentheses) (see spectra in **Figure 7** and conditions in Material and Methods).

	**Softwood lignin**		**Hardwood lignin**	
	Control	ApeLiP	Control	ApeLiP
a) Lignin composition and decay[a]				
Guaiacyl units (**G**)	126 (100)	36 (71)	12 (100)	1 (92)
Syringyl units (**S**)	0	0	27 (100)	5 (82)
Total lignin	126 (100)	36 (71)	39 (100)	6 (85)
Lignin S/G ratio	0	0	2.2	4.7
b) Side-chain inter-unit linkages[b]				
β-*O*-4′ ethers (**A**)	87 (33)	90 (28)	88 (31)	89 (28)
Phenylcoumarans (**B**)	9 (4)	6 (2)	4 (1)	4 (1)
Resinols (**C**)	4 (2)	4 (1)	8 (3)	7 (2)
Total	100 (38)	100 (31)	100 (35)	100 (32)

[a]Relative intensities (DMSO signal= 100) of G and S signals (with decay percentages in parentheses); [b]Percentage (A+B+C= 100) of lignin substructures with different side-chain linkages (with values referred to total lignin units [G+S= 100] in parentheses).

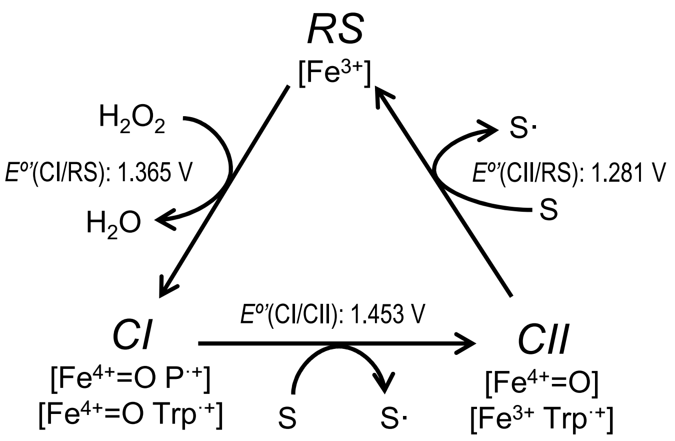

Figure S1. Catalytic cycle of ligninolytic peroxidases and E°′ of the ApeLiP CI/RS, CI/CII and CII/RS pairs estimated in the present study. The catalytic cycle includes: **i)** Resting state (RS; Fe^{3+}) activation by H_2O_2 yielding CI, that retains two oxidizing equivalents in the form of Fe^{4+}=O and porphyrin cation radical ($P^{\bullet}$+), the latter in equilibrium with Fe^{4+}=O and tryptophanyl cation radical ($Trp^{\bullet}$+); **ii)** CI reduction to CII, bearing only one oxidation equivalent on the Fe^{4+}=O (in equilibrium with Fe^{3+} $Trp^{\bullet}$+), during substrate (S) oxidation (to $S^{\bullet}$ radical); and **iii)** CII reduction to RS during second substrate oxidation.

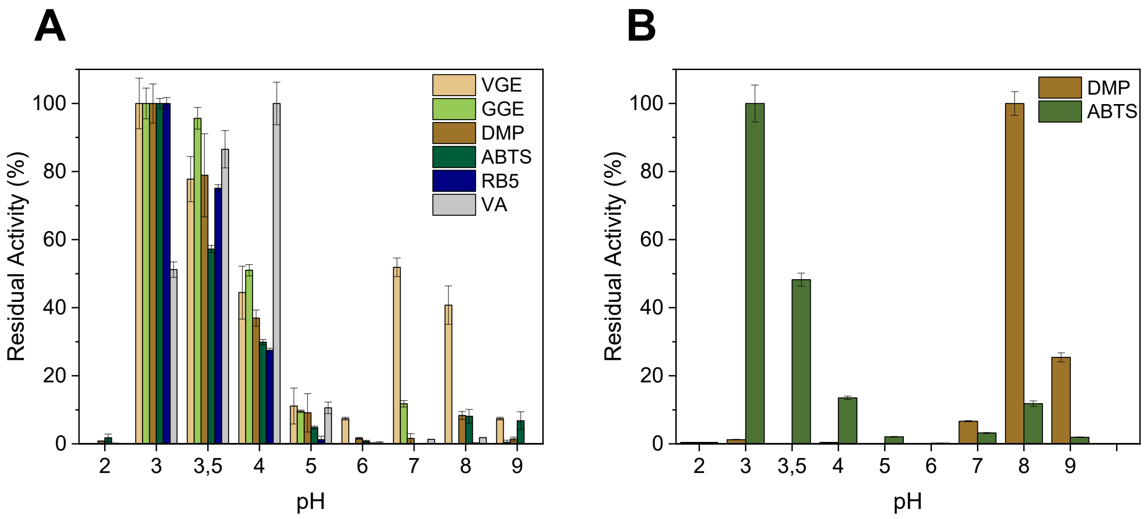

Figure S2. Optimal pH for the oxidation of high (VGE, RB5, VA) and low (GGE, DMP, ABTS) redox-potential substrates by ApeLiP (**A**) and its W166A variant (**B**). Reactions were initiated by H_2O_2 (0.1 mM) and activities were measured at pH 2-9 in 100 mM Britton-Robinson buffer. Residual activities (means and standard deviations) were expressed as percentages of the maximal activity for each substrate. The pH optimum for dimer (VGE and GGE) oxidation was only analyzed with ApeLiP. No oxidation of the high redox-potential substrates was observed with the W166A variant.

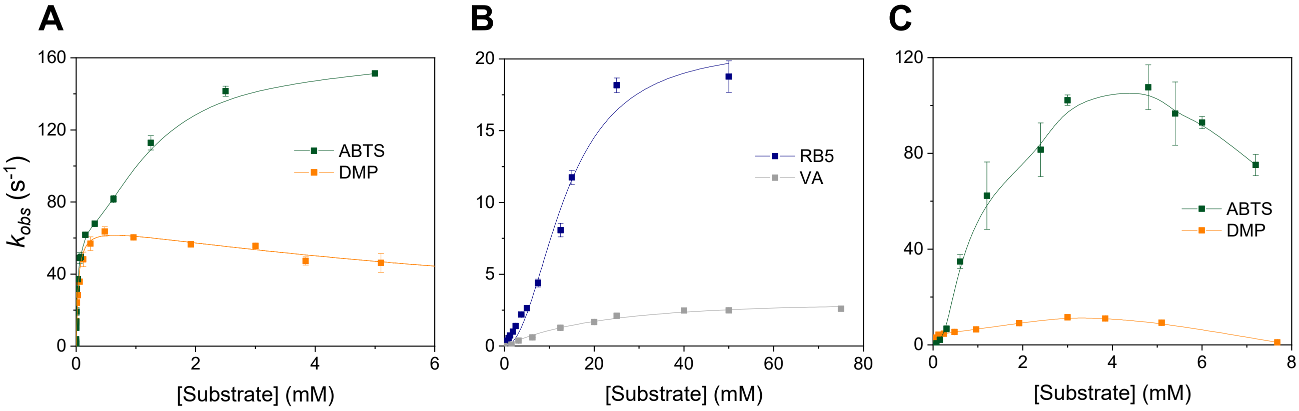

Figure S3. Kinetic curves for substrate (ABTS, DMP, RB5 and VA) oxidation by ApeLiP **(A, B)** and its W166A variant **(C)**. Reactions were measured at 25°C in 100 mM sodium tartrate or Tris-HCl at the optimal pH (see **Fig. S2**) and saturating H_2O_2 concentrations (0.4 and 1 mM for ApeLiP and its W166A variant, respectively). Means and standard deviations are shown.

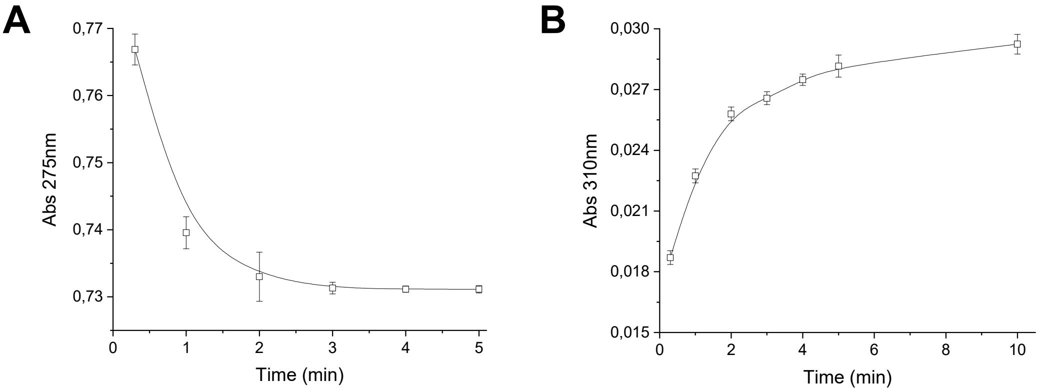

Figure S4. Time courses of ApeLiP reaction with GGE (**A**) and VGE (**B**) lignin model dimers. 0.25 mM GGE oxidation by 0.1 µM enzyme was followed during 5 min by absorbance decrease at 275 nm. 1 mM VGE oxidation by 0.8 µM ApeLiP was followed during 10 min by absorbance increase at 310 nm due to veratraldehyde formation. Reactions were performed in 100 mM sodium tartrate at optimal pH 3.0 and started by addition of 0.4 mM H_2O_2. Means and standard deviations are shown.
